# Significance analysis of microarray for relative quantitation of LC/MS data in proteomics

**DOI:** 10.1186/1471-2105-9-187

**Published:** 2008-04-10

**Authors:** Bryan AP Roxas, Qingbo Li

**Affiliations:** 1 Center for Pharmaceutical Biotechnology, University of Illinois at Chicago, Chicago, IL 60607, USA; 2 Department of Microbiology and Immunology, University of Illinois at Chicago, Chicago, IL 60607, USA

## Abstract

**Background:**

Although fold change is a commonly used criterion in quantitative proteomics for differentiating regulated proteins, it does not provide an estimation of false positive and false negative rates that is often desirable in a large-scale quantitative proteomic analysis. We explore the possibility of applying the Significance Analysis of Microarray (SAM) method (PNAS 98:5116-5121) to a differential proteomics problem of two samples with replicates. The quantitative proteomic analysis was carried out with nanoliquid chromatography/linear iron trap-Fourier transform mass spectrometry. The biological sample model included two *Mycobacterium smegmatis *unlabeled cell cultures grown at pH 5 and pH 7. The objective was to compare the protein relative abundance between the two unlabeled cell cultures, with an emphasis on significance analysis of protein differential expression using the SAM method. Results using the SAM method are compared with those obtained by fold change and the conventional *t*-test.

**Results:**

We have applied the SAM method to solve the two-sample significance analysis problem in liquid chromatography/mass spectrometry (LC/MS) based quantitative proteomics. We grew the pH5 and pH7 unlabelled cell cultures in triplicate resulting in 6 biological replicates. Each biological replicate was mixed with a common ^15^N-labeled reference culture cells for normalization prior to SDS/PAGE fractionation and LC/MS analysis. For each biological replicate, one center SDS/PAGE gel fraction was selected for triplicate LC/MS analysis. There were 121 proteins quantified in at least 5 of the 6 biological replicates. Of these 121 proteins, 106 were significant in differential expression by the *t*-test (*p *< 0.05) based on peptide-level replicates, 54 were significant in differential expression by SAM with Δ = 0.68 cutoff and false positive rate at 5%, and 29 were significant in differential expression by the *t*-test (*p *< 0.05) based on protein-level replicates. The results indicate that SAM appears to overcome the false positives one encounters using the peptide-based *t*-test while allowing for identification of a greater number of differentially expressed proteins than the protein-based *t*-test.

**Conclusion:**

We demonstrate that the SAM method can be adapted for effective significance analysis of proteomic data. It provides much richer information about the protein differential expression profiles and is particularly useful in the estimation of false discovery rates and miss rates.

## Background

Fold change is commonly used in quantitative proteomic analysis where proteins differing by more than an arbitrary cut-off value in abundance are considered to be differentially expressed [1-5]. A fold change test is equivalent to a global *t*-test assuming homogenous variance between different proteins. Although it is a convenient and cost effective way to evaluate protein expression level differences between two conditions, fold change alone is not a statistical test that can indicate the level of confidence in differential expression of proteins.

Rapid development of liquid chromatography-mass spectrometry (LC/MS) based proteomics has led to gradual replacement of the traditional 2D gel approach by the LC/MS approach. Accordingly, variation and quality control of quantitation by LC/MS has been actively explored [6-12]. The importance of significance analysis for biomarker discovery has also been stressed [13]. Molina *et al*. simultaneously measured three states of Hela cells in response to stimuli using SILAC labeling for quantitation [6]. Fold changes were evaluated at protein and peptide level by analysis of variance performed in the statistical program R. The authors demonstrated the capability of detecting 1.8-fold change at a significance level of 95%. No significance score was assigned to individual proteins, however. Piening *et al*. proposed Mass Deviance, a quality control metric, for assessing the accuracy of peptide detection in *Saccharomyces cerevisiae *[7]. This approach was rigorous at validating peptide identification in LC/MS but not yet directly applicable for quantifying relative abundance. Meng *et al*. used the differential mass spectrometry (dMS) method for label-free LC/MS profiling, demonstrating detection of peptides with a change as small as 1.5-fold with ~20% relative errors in peptide relative abundance in a processed plasma background [8]. Andreev *et al*. developed Q-MEND algorithm for label-free quantitation of relative protein abundances across multiple complex *E. coli *proteome samples, achieving 7% quantitation accuracy and mean precision of 15% [9]. Wang *et al*. reported the algorithm Quoil for label-free quantitation measurements across repeated LC/MS runs with Student's *t*-test after applying the step-down adjustment of probability threshold [14]. Most recently, reproducibility assessment of differential quantitation by SILAC, ICAT, and label-free methods was reported [11]. In this study, a ratio distribution analysis was applied to common peptides between samples to remove outliers until a normal distribution was obtained. Using the filtered common peptides, it was assessed that 95% of the total common peptides have intensities within a ~2-fold change for a pair of cultures of T47D human breast cancer cells, with SILAC analysis having the best summary statistics. These conclusions were drawn from peptide-level quantitation in combination with a ratio distribution analysis. Earlier efforts in statistical and computational methods for quantitative proteomics by LC/MS was reviewed by Listgarten and Emili [12]. These studies showed a great deal of efforts and progress in statistical analysis of LC/MS data in proteomics. Currently, few have systematically assessed significance of analysis at a systems level along with estimation of false positive and negative rates.

In this work, we explore the use of the Significance Analysis of Microarray (SAM) method [15] for analysis of a two-sample significance problem in LC/MS quantitative proteomics. We used *Mycobacterium smegmatis *cells grown at pH 5 and pH 7 in unlabeled media as the two-sample model. We also grew one ^15^N-labeled *M. smegmatis *cell culture and used it as the internal standard to normalize the protein abundance in the pH5 and pH7 unlabeled cells by the popular ^14^N/^15^N quantitation method [16]. Cell protein extracts were first fractionated by SDS/PAGE. Then a high resolution nanoliquid chromatography/linear ion trap-Fourier transform mass spectrometry (nanoLC/LTQ-FTMS) system was used for peptide separation and identification. The LC/MS data was further quantified by the previously described algorithm [10]. We report the results of quantifying the protein relative abundance between the pH5 and pH7 unlabeled cells, with an emphasis on significance analysis of protein differential expression using the SAM method in comparison with fold change and conventional *t*-test methods.

## Results and Discussion

In this study, we report using the SAM method to solve the two-sample significance analysis problem in LC/MS based quantitative proteomics. We compare the SAM method with the conventional fold change test and *t*-tests.

SAM was originally developed for microarray analysis by Tusher *et al*. [15]. Development of this method was initially propelled by the need to resolve the issue of multiplicity of testing in conventional *t*-tests when a large number of genes were studied simultaneously. As Tusher *et al*. stated, "SAM identifies genes with statistically significant changes in expression by assimilating a set of gene specific *t*-tests. Each gene is assigned a score on the basis of its change in gene expression relative to the standard deviation of repeated measurements for that gene. Genes with scores greater than a threshold are deemed potentially significant with an assigned *q*-value." SAM incorporated *q*-value as a measurement of the significance of a gene based on the work of Storey [17]. Each time when the threshold is adjusted, a false discovery rate is estimated for the resulting set of genes with significant differential expression. Low-level data processing in the LC/MS measurements is typically very different from that in DNA microarray experiments. However, at the higher level of protein differential expression determination, we treated the protein abundance data the same as the gene abundance data and used the SAM method without modification.

For statistical analysis in this work, the pH5 and pH7 *M. smegmatis *cell cultures were grown in triplicate resulting in total 6 biological replicates. We also grew a ^15^N-labeled *M. smegmatis *culture and used it as the reference for normalizing the 6 unlabeled biological replicates. Each of the 6 unlabeled biological replicates was first mixed with the ^15^N-labeled reference. It was then processed for protein quantitation by the widely used ^14^N/^15^N relative abundance measurement method [10,16]. After all 6 unlabeled biological replicates were normalized to the ^15^N-labeled reference, they were analyzed either by fold change test, *t*-test or the SAM method.

In the following sections, we discuss the experimental layout of sample replicates, fold change analysis, random fluctuation of measurements, conventional *t*-tests, SAM analysis, and differentially expressed proteins.

### Sample replicates

In DNA microarray experiments, arrays are often spotted with gene probes in replicates. The typical practice is to average the replicates for each probe before assessing the differential expression of the gene. Since the geometrical arrangement of gene probes on an array is known before an experiment, the replicates for a gene can be known *a priori *within an array and across multiple arrays. In proteomics, assignment and cross-reference of peptides and proteins across multiple LC/MS analysis is not as straightforward. In a typical LC/MS based proteomics experiment, a protein is digested with an enzyme into multiple peptides. The mixture of peptides from multiple proteins is injected into a LC/MS instrument for separation and peptide identification by MS/MS scan. Protein relative abundance is assessed from the quantitation of one or more peptides originating from the protein. This process is analogous to the quantitation of genes based on multiple gene probes.

Contrary to DNA microarray experiments, one distinct characteristic of LC/MS based quantitative proteomics is that the number of peptides being quantified is usually not known *a priori*. There are different reasons for this. For example, if a peptide is highly hydrophobic or highly negatively charged, the chance of identifying this peptide by LC/MS is significantly reduced. There are also LC/MS instrument related considerations. A data-dependent acquisition algorithm is employed in most LC/MS instrument methods for peptide identification [18]. Due to the limited speed with which a mass spec can acquire MS/MS spectra for peptide identification, only a limited number of precursor ions with the highest intensities in one MS scan will be selected for MS/MS identification. Currently the sampling rate of a typical LC/MS instrument can easily be overwhelmed by the complexity of a protein sample. Saturation of sampling a complex protein mixture requires more than just a few replicate runs. For this reason, a common way to increase the number of identified and quantified peptides for a sample is to pool the peptides identified by MS/MS from replicate runs of the same sample [5].

Callister *et al*. [19] demonstrated an accurate mass and time tag (AMT) approach to overcome the above mentioned limitation. In this approach, the LC/MS scanning process is decoupled from the MS/MS peptide sequencing process. This is done by first accumulating enough MS/MS identification of peptides followed by high-throughput LC/MS analysis. This powerful AMT approach skips the rate limiting MS/MS step. It thus avoids the random sampling effect of the MS/MS peptide identification process. However, successful application of this approach relies upon a database containing the AMTs accumulated from multiple LC/MS/MS runs. This requires precise control of the LC/MS/MS and LC/MS operation parameters to ensure the reliability of AMTs.

Because the primary focus of this work was to compare several statistical significance analysis methods, we opted to take a straight-forward approach by only quantifying those peptides with confident MS/MS identification. These were the peptides assigned a probability of misidentification smaller than 0.01 by the BioWorks software based on a MS/MS spectrum database search. A probability of 0.01 implies one misidentification out of 100 by chance. A peptide may be identified at different charge states typically ranging from +1 to +4. The most often observed charge state is +2 or +3 in the nanoLC/LTQ-FTMS system. BioWorks assigns a probability for a peptide detected at each charge state. Accordingly, a peptide detected at a particular charge state is called a peptide charge state (PCS) [10].

In this study, we grew the pH5 and pH7 unlabelled cell cultures in triplicate resulting in a total of 6 biological replicates. Each biological replicate was mixed with the ^15^N-labeled reference sample prior to SDS/PAGE fractionation and LC/MS analysis. Each biological replicate was analyzed by nanoLC/LTQ-FTMS with triplicate runs. The PCS's with *p *< 0.01 from the triplicate runs of a biological replicate were combined for calculating the protein and peptide relative abundance [10]. Statistical analysis of relative abundance between the pH5 and pH7 unlabelled cells was performed for the proteins quantified in at least 5 of the 6 replicates. The 6 biological replicates were designated as pH5A, pH5B, pH5C, pH7A, pH7B, and pH7C (Table 1). The average of the pH5 biological triplicates was named pH5av. Similarly, the average of the pH7 biological triplicates was named pH7av.

**Table 1:** Sample names. The first number in parenthesis is the number of quantified proteins for the sample replicate name preceding the two numbers in the parenthesis. The second number in the same parenthesis is the average number of PCS's quantified for a protein in the same sample replicate. See main text for more details.

Cell sample	Culture triplicates	*in silico *pooled replicate	Average of culture triplicates
pH5	pH5A (119, 15)	pH5p (174, 41)	pH5av
	pH5B (120, 14)		
	pH5C (121, 15)		
pH7	pH7A (112, 14)	pH7p (174, 31)	pH7av
	pH7B (110, 14)		
	pH7C (113, 13)		

In addition, we pooled the PCS's from the pH5 biological triplicates to calculate the protein relative abundance for the *in silico *pooled replicate for the pH5 cells, which we called pH5p. Similarly, we also pooled the PCS's from the pH7 biological triplicates to calculate the protein relative abundance for pH7p, the *in silico *pooled replicate for the pH7 cells (Table 1).

The protein mixture of each biological replicate was fractionated into 5 fractions by SDS/PAGE. Only the center fraction was further processed for nanoLC/LTQ-FTMS analysis. Although it was desirable to analyze all the fractions in triplicate LC/MS analysis, we chose to only analyze the center fraction for each biological replicate for two reasons. First, focusing on one common fraction for all 6 biological replicates is sufficient for demonstrating the principle of statistical analysis we investigated in this work. Second, we were conservative about the cost of analyzing all 5 fractions for all 6 replicates because it would have required 90 LC/MS runs lasting for more than 135 hrs. This estimation was based on 5 SDS/PAGE fractions per biological replicate, triplicate runs per SDS/PAGE fraction, and 90 min per run (see Methods).

**Figure 1:**
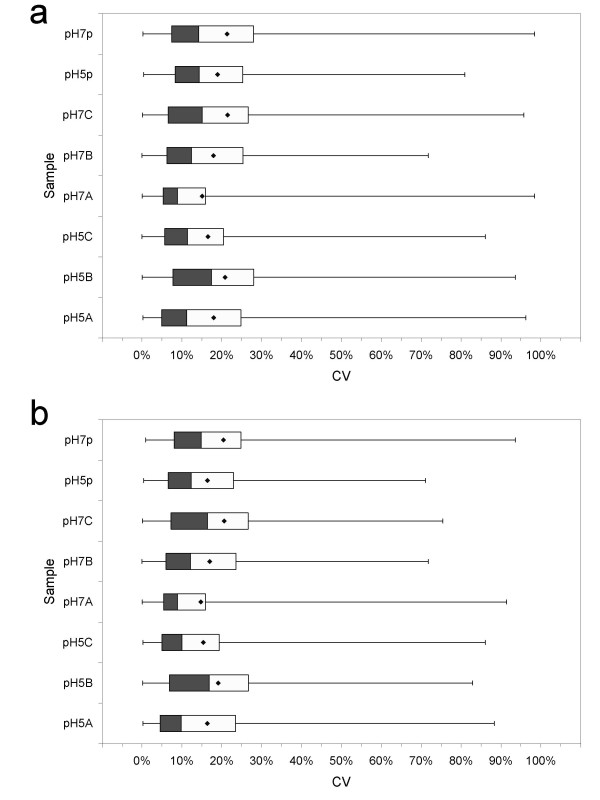
**CV summary statistics**. Boxplots displaying the summary statistics of the coefficient of variance (CV) of protein relative abundances for the pH5 culture triplicates (pH5A, pH5B, and pH5C), the pH7 culture triplicates (pH7A, pH7B, and pH7C), and the *in silico *pooled replicates (pH5p and pH7p). A boxplot summarizes the minimum, 25 percentile, 50 percentile, 75 percentile, and maximum CV's of a sample. a) Boxplots are plotted for the 174 proteins quantified between pH5p and pH7p. These 174 proteins include all those proteins quantified in pH5A(159), pH5B(159), pH5C(161), pH7A(131), pH7B(124), and pH7C(134). The numbers in parenthesis indicate the number of protein in a sample. b) Boxplots are plotted for the 121 proteins quantified in at least 5 of the 6 replicates of the pH5 and pH7 culture samples. The diamond dots indicate the mean CV's.

With only the center fraction analyzed, there were 121 proteins quantified in at least 5 of the 6 biological replicates of the pH5 and pH7 samples (Table 1). Ninety were quantified in all 6 replicates, and 31 in 5 replicates. Figure 1a shows the CV boxplots for these proteins in the 6 biological replicates and the 2 *in silico *pooled replicates. Meanwhile, there were 174 proteins found in common between pH5p and pH7p. The CV boxplots for these 174 proteins are shown in Figure 1b. The complete set of protein and peptide data for statistical significance analysis is summarized in Table 2 (see Additional file 1). Table 2 shows the protein relative abundance, standard deviation, number of unique peptides and number of PCS's for each protein in the sample replicates pH5A, pH5B, pH5C, pH7A, pH7B, pH7C, pH5p, and pH7p. Results of the fold change test, the 2 *t*-tests, and the SAM analysis to be described in a later section are shown to the right of Table 2.

Figure 1 indicates that more than 75% of proteins in every sample replicate have CV less than 30%. The mean CV and median CV for all sample replicates was less than 21% and 15%, respectively. It was noticed that the CV summary statistics were improved only slightly for the 121 proteins in Figure 1a compared to the 174 proteins in Figure 1b. The average of the average CV's for the biological triplicates was 18 ± 2% for the pH5 sample, and 18 ± 3% for the pH7 sample. For the 174 proteins common between pH5p and pH7p, the average CV was 19% and 21% for pH5p and pH7p, respectively. These results indicate that the sample replicates have consistent CV summary statistics. They are suitable for use in subsequent analysis to compare several significance analysis methods.

### Fold change analysis

Since pH5p and pH7p represent the average of both analytical and biological replicates for the pH5 and pH7 unlabeled culture samples respectively, we examine the number of differentially expressed proteins between the two samples by the fold change test. Within this context, fold change refers to the ratio of relative abundance of a protein between the pH5 and pH7 unlabeled samples. It has a value greater than or equal to 1. This definition is consistent with that of the SAM.

Based on the simple 2- and 3-fold change tests, 55 and 29 proteins were respectively found to be differentially expressed between pH5p and pH7p (Table 2). As discussed earlier, the fold change threshold alone is not a statistical test that can indicate the level of confidence about differentially expressed proteins. It does not reveal the random fluctuation inherent in protein differential expression levels. It would be of interest to test the level of such random fluctuation. As described below, we took a simple approach to test if random fluctuation was confined within a 2- or even 3-fold change boundary.

### Random fluctuation

**Figure 2:**
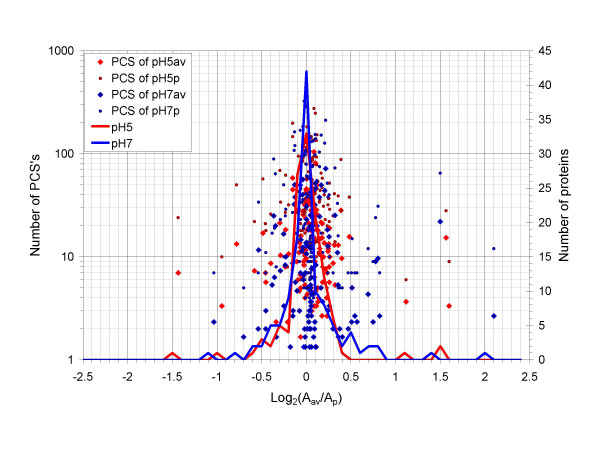
**Random fluctuation**. The number of quantified PCS's of a protein is plotted against the log ratio of the average relative abundance of its biological triplicates (A_av_, representing either A_pH5av _or A_pH7av_) over the relative abundance of its *in silico *pooled replicate (A_p_, representing either A_pH5p _or A_pH7p_) for the pH5 and pH7 samples respectively. The red diamonds and the small red dots represent the pH5 sample. The blue diamonds and the small blue dots represent the pH7 sample. The diamond symbols represent the average number of PCS's of a protein of the biological triplicates. The small dots represent the number of total PCS's of a protein in the *in silico *pooled replicates. The histograms based on the number of quantified proteins are also plotted, with the red trace representing the pH5 sample and the blue trace representing the pH7 sample.

To test random fluctuation, the number of quantified PCS's of a protein was plotted against the log ratio between the average of relative abundance of its biological triplicates (A_av_, representing either A_pH5av _or A_pH7av_) and the relative abundance of its *in silico *pooled replicate (A_p_, representing either A_pH5p _or A_pH7p_), as shown in Figure 2. In addition, the histogram for each sample was also plotted based on protein number and log_2_(A_av_/A_p_). We reasoned that pH5av *versus *pH5p or pH7av *versus *pH7p represents a form of permutation for the biological triplicates of the pH5 or pH7 sample. The distribution of log_2_(A_av_/A_p_), as summarized by the histograms, should therefore reveal some random errors in protein relative abundance quantitation. We chose the number of PCS's for plotting against log_2_(A_av_/A_p_) because it was interesting to examine the effect on random errors.

From Figure 2, it was noted that most of the proteins clustered within 1.5 fold change, or ± 0.585 on the log base 2 scale. The 95% interval was -0.52 to 0.48 for the pH5 sample (red trace) and -0.53 to 0.80 for the pH7 sample (blue trace). There were 3 proteins in the pH5 sample and 4 in the pH7 sample falling outside the 2-fold boundary. There was 1 protein in the pH5 sample and 1 in the pH7 sample falling outside the 3-fold boundary. There were 3 proteins in the pH5 sample and 2 in the pH7 sample falling within the range of 2- to 3-fold change. There were 2 proteins in the pH5 sample and 6 in the pH7 sample falling within 1.5- to 2-fold change range.

These results suggested that the random errors could occur outside a 2- or even 3-fold change boundary. In addition, the random errors shown in Figure 2 were not limited to those proteins that had a very low number (< 5) of PCS's, even though the trend was that random errors mostly occurred below 25 PCS's for pH5av and pH7av or below 70 for pH5p and pH7p.

To evaluate the influence of random fluctuation on the confidence of measured protein differential expression, we performed 2 *t*-tests as described in the following. One *t*-test was based on peptide-level replicates. The other was based on protein-level replicates.

### *t*-tests

In general, a *t*-test is used to evaluate whether the means of control and experiment groups are statistically different. The *t*-value is the ratio between the difference of group means and the variability of groups. The standard deviation of the *t *distribution is determined by the number of degrees of freedom derived from the sample sizes. The number of degrees of freedom need not be the same for the control and the experiment groups. For the same *z *score, a falling sample size will make the *t *distribution take on an increasingly larger standard deviation. Increased standard deviation of the *t *distribution has the tendency to incur a higher false negative rate. On the other hand, a very large number of degrees of freedom may allow a higher false positive rate. In this analysis, the number of degrees of freedom may be very high for pH5p and pH7p for some proteins when it is based on the number of PCS's detected for each protein, i.e., peptide-level replicates. However, the number of degrees of freedom is no more than 3 when protein-level replicates are used. In either case, the *t*-tests do not require equal number of degrees of freedom between control and experiment.

For simplicity in describing proteins found to have statistically significant differential expression, the term "significant protein" is used hereafter with the meaning of "protein with significant differential expression".

#### t-test with peptide-level replicates

To test if the observed differential expression of these proteins was significant, a two-sample *t*-test assuming equal variances was performed on the 174 proteins using peptide-level relative abundance information. We adopted the *t*-test which was previously demonstrated by Wu *et al*. [20] in quantitative proteomic analysis of mammalian organisms. To compute the two-sample *t*-test, a pooled standard deviation was first calculated from the standard deviations of the protein relative abundance of the 2 samples. The pooled standard deviation was between the 2 standard deviations with greater weight given to the standard deviation of the sample with larger number of PCS's detected. The mathematical formula for the *t*-test was fully described by Wu *et al*. [20]. Since all of the PCS's were pooled to calculate the protein relative abundance in pH5p and pH7p, there was only one protein relative abundance value for each protein in pH5p or pH7p. The *t*-test for comparing pH5p and pH7p was thus performed using peptide-level replicates without protein-level replicates. This means that the number of degrees of freedom for measuring a protein was represented by multiple PCS measurements for that protein. This *t*-test with peptide-level replicates is different from that described later with protein-level replicates.

**Figure 3:**
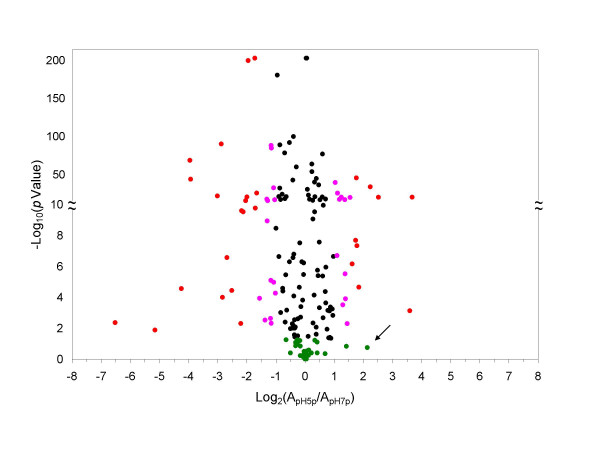
**Volcano plot for pH5p and pH7p**. Volcano plot for the *in silico *pooled replicates pH5p and pH7p. The green dots represent the proteins found insignificant (*p *>= 0.05) by the *t*-test based upon the protein relative abundances of pH5p and pH7p and the number of PCS's used for quantifying each protein. The black, pink, and red dots represent the significant proteins with fold change of less or equal to 2, greater than 2 but lees or equal to 3, and greater than 3 respectively. The arrow indicates the protein with 3-fold change but found not significant by the *t*-test. See text for more details.

We used the volcano plot in Figure 3 to visualize the proteins categorized as up- or down-regulated based on the simple 2- and 3-fold change thresholds, and to display their statistical significance based on the *t*-test with peptide-level replicates. In the volcano plot, the *t*-test *p *value was plotted against the relative abundance ratio between pH5p and pH7p on a logarithmic scale. The *t*-test rejected one of the proteins found upregulated in pH5p with greater than 3-fold change (the green dot with an arrow). This resulted in a total of 53 proteins having greater than 2-fold change with *t*-test significance (*p *< 0.05). Of these 53 proteins, 25 had fold change between 2 and 3 (pink dots), and 28 had greater than a 3-fold change (red dots). Of the remaining 120 proteins that had less than a 2-fold change, 32 were not significant (*p *>= 0.05, green dots), and 88 were significant (*p *< 0.05, black dots).

#### t-test with protein-level replicates

The above *t*-test with peptide-level replicates utilized the PCS's of a protein quantified in pH5p and pH7p. Each PCS should be an independent event for a protein. This assumption is complicated by several factors. In LC/MS based proteomic experiments, detection of a PCS depends not only on its concentration but also on the composition of the peptide mixture. Ion suppression effect in electrospray ionization, space charge effect in FT mass spectrometer, LC column separation efficiency for complex samples, and data-dependant acquisition, etc., can directly or indirectly affect the quantitation of a PCS. Therefore, a conventional *t*-test performed on such data requires cautious interpretation.

For comparison, we performed the second *t*-test at a protein level. This means that only protein relative abundance values were used without referring to the PCS information as for the *t*-test shown in Figure 3. Basically, pH5A, pH5B, and pH5C represented the triplicates for the pH5 sample. pH7A, pH7B, and pH7C represent the triplicates for the pH7 sample. Using the 2 sets of protein-level triplicates, we calculated their respective average pH5av and pH7av. To perform the *t*-test, the 2 sets of protein-level replicates were input as two arrays in the Microsoft Excel TTEST function with option selection of two-sample equal variance, two-tailed, and type of homoscedastic.

**Figure 4:**
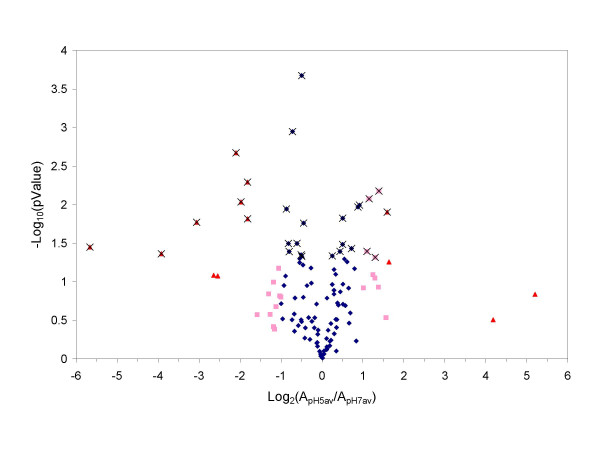
**Volcano plot for pH5av and pH7av**. The blue diamonds, pink squares, and red triangles represent proteins with fold change of less or equal to 2, greater than 2 but lees or equal to 3, and greater than 3 respectively. The x marks indicate that a protein is found significant (*p *< 0.05) by the *t*-test based on triplicate protein relative abundances without referring to peptide information. See text for more details.

Figure 4 shows the volcano plot for the protein relative abundance ratios between the pH5 and pH7 samples based on the triplicate protein relative abundances for each sample. Compared to Figure 3, one apparent difference is that a smaller number of significant proteins (55%) had fold change less than 1.5 (0.585 on log base 2 scale). The percentage of significant proteins was also reduced to 24% in Figure 4 compared to 81% in Figure 3. Of the 14 proteins with greater than 3-fold change, 9 (69%) were tested significant, compared to 28 out of 29 (97%) in Figure 3.

There is an apparent discrepancy between the *t*-test results shown in Figure 3 and Figure 4 based on respective peptide- and protein-level information. This suggests that we need a third method to examine whether the two conventional *t*-test methods are overly aggressive or conservative. To do so, we need to assess not only the individual protein significance but also the false positive and false negative rates for the group of proteins under significance testing. A similar issue has been extensively investigated in DNA microarray data analysis. SAM is one of the widely accepted methods for such analysis in DNA microarray. In the following, we explore the applicability of the SAM method towards our proteomics problem.

### Significance analysis with SAM

As described earlier, SAM is a statistical technique originally developed for finding genes with significant differential expression in a set of microarray experiments [21]. SAM is capable of taking input from different response variables. For our proteomics problem of two-sample significance analysis between the pH5 and pH7 cell cultures, the response variable is equivalent to a grouping of untreated (pH7) and treated (pH5) samples (unpaired). For each sample, at least two replicates are required by SAM. Using the protein-level replicates from the pH5 and pH7 samples, SAM calculates observed and expected scores for each protein. The observed score represents the relative difference of a protein between the pH5 and pH7 samples. The expected score represents the random fluctuation when there is no difference between the two samples. When the difference between the observed and expected scores is beyond a certain threshold, the protein is called significant in differential expression.

To perform the SAM analysis, the protein relative abundance data from the pH5 and pH7 biological triplicate samples were input as two-class unpaired response type into SAM, with 600 permutations, *t*-statistic test, 1% fixed percentile for estimation of s0 factor for denominator, and K-nearest neighbors imputer as the imputation engine with 5 neighbors for filling missing values. Due to the space limit here, we will not repeat further detail description of the SAM software and its operation. The users guide and technical documents for SAM are readily available elsewhere by Chu *et al*. [21]. We will instead focus on the result output and interpretation of the method.

**Figure 5:**
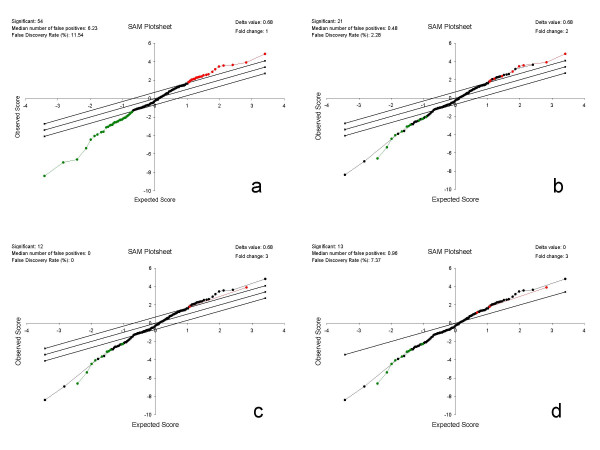
**SAM plotsheet outputs**. SAM plotsheet outputs under the four sets of criteria: a) Δ = 0.68, fc = 1; b) Δ = 0.68, fc = 2; c) Δ = 0.68, fc = 3; d) Δ = 0, fc = 3, which are indicated at the upper right corner of each plotsheet. The red, green, and black dots represent upregulated, downregulated, and insignificant proteins respectively. The upper and lower 45° degree lines indicate the Δ threshold boundaries. Proteins with Δ = 0 would fall on the 45° line through the origin. The number of significant proteins, median number of false positives, and false discovery rate are indicated at the upper left corner of each plotsheet.

Figure 5 shows the results from the SAM analysis. The SAM plotsheets are presented with slight graphical modification, with the Δ value and fold change inserted into the upper right corner for convenience of comparison. Each SAM plotsheet contains all the proteins plotted by their observed scores and expected scores.

The observed score is the relative difference [15] in protein expression. It is calculated by dividing the difference between protein relative abundances in the pH5 and pH7 samples by the pooled standard error of repeated measurements of that protein in the pH5 and pH7 samples [15]. The expected score is calculated using the large set of permutations of protein relative abundance data of the 6 biological replicates from the pH5 and pH7 samples.

The observed score provides a control over random fluctuation, while the expected score allows assignment of statistical significance. The correlation of these two scores is used for identifying proteins with potentially significant differential expression as shown in Figure 5. If a protein has absolutely no differential expression, the observed relative difference would be the same as the random fluctuation that is represented by the expected score. The data point of such a protein in the SAM plotsheet would fall on the 45° line through the origin. Data points representing differentially expressed proteins will deviate from this 45° line. The point displacement of a protein from the 45° line through the origin is quantitatively measured by a Δ value in SAM. Proteins with Δ values beyond a certain threshold are called significant. The 45° upper and lower Δ lines indicate the boundary defined by a selected Δ value.

SAM provides an estimation of false discovery rate (FDR) for the proteins called significant by each Δ value. A Δ value can be set together with a fold change threshold. FDR is calculated from the average number of falsely significant proteins in all the permutations divided by the number of proteins called significant above that Δ threshold.

Figure 5a presents the results with Δ = 0.68. This Δ value results in 6.23 estimated false positives out of the 121 proteins under testing, equivalent to a 5.1% false positive rate. This is the same as the nominal false positive rate defined by a *p *< 0.05 threshold in a conventional *t*-test. Fifty-four proteins are called significant with a FDR of 11.54%, with 22 upregulated and 32 downregulated in the pH5 *versus *the pH7 sample.

Combination of Δ = 0.68 with 2-fold change reduces the number of significant proteins to 21 with a FDR of 2.28% (Figure 5b). When Δ = 0.68 is used together with 3-fold change, the number of significant proteins are further reduced to 12, with a FDR of 0 (Figure 5c). When the 3-fold change criterion is used alone, there are 13 proteins called significant with a FDR of 7.37% (Figure 5d). The 13^th ^protein (MSMEG4520) increases the total significant proteins determined by SAM using the four different criteria to 55, as shown in Figure 5. For comparison, Table 3 lists these 55 proteins with their analysis output by SAM and the 2 conventional *t*-tests (see Additional file 2).

Of the 55 proteins, 26 were found significant by the *t*-test (*p *< 0.05) performed on the triplicates of the pH5 and pH7 samples (Figure 4), and all were found significant by the *t*-test (*p *< 0.05) performed on the *in silico *pooled replicates of the pH5 and pH7 samples (Table 2). Of the 13 proteins with greater than 3-fold change in Table 3, only 9 were found significant by the *t*-test shown in Figure 4. It is noted that there are 14 proteins with greater than 3-fold change shown in Figure 4. This extra 14^th ^protein (MSMEG2382) with greater than 3-fold change in Figure 4 has a significant fold change of 2.8 calculated by SAM after imputation. This protein was not found significant under the *t*-test in Figure 4, even though it showed a fold change of 3.1 in Figure 4. Of the 121 proteins analyzed by SAM, 106 are called significant by the *t*-test (*p *< 0.05) shown in Figure 3, whereas only 54 are called significant by SAM with Δ = 0.68 cutoff which controls false positive rate at 5% and FDR at 13.1%. Therefore, the *t*-test for the *in silico *pooled replicates pH5p and pH7p shown in Figure 3 is overly aggressive while the *t*-test in Figure 4 appears to be overly conservative. These results indicate that SAM provides more reasonable results. The resampling approach used by SAM appears to overcome the false positives one encounters using the peptide-based *t*-test while allowing for identification of a greater number of differentially expressed proteins than the protein-based *t*-test.

Most importantly, for each significant protein, SAM assigns a *q*-value that represents the minimum FDR of the list of proteins having Δ values and/or fold changes equal to or greater than that at which the protein is called significant in differential expression. Therefore, *q-*value quantitatively measures how significantly the protein is differentially expressed. This is the lowest FDR at which the protein is called significant [17,21]. As further explained by Chu *et al*. [21], it is like the familiar '*p*-value' but adapted to the analysis of a large number of genes. In other words, it is the *p-*value at which proteins with Δ values and/or fold changes smaller than the significant threshold are actually differentially expressed. The *q*-values for proteins called significant under different Δ and/or fold change criteria are presented in Table 3 in comparison with conventional *t*-tests.

Thirty-four (63%) of the 54 proteins called significant with the Δ = 0.68 threshold have fold change between 1.2 and 2.0. Of these 34 proteins, 22 (65%) have a *q*-value greater than 5%. For the 20 (37%) proteins with greater than 2-fold change, 3 (15%) have a *q*-value greater than 5% (Table 3). This illustrates that *q*-value properly predicts the significance of protein differential expression. While conventional *t*-tests provide an estimation of probability for individual proteins, the distribution of errors is not known.

Combination of Δ = 0.68 and 2-fold change results in 21 significant proteins, of which 15 have a *q*-value of 0 and 6 have a *q*-value between 2.3 and 3.0. Combination of Δ = 0.68 and 3-fold change results in 12 significant proteins all of which have a *q*-value of 0. Using the 3-fold change criterion alone generates 13 significant proteins that include the 12 proteins called significant by Δ = 0.68 and 3-fold change. The 13^th ^additional protein (MSMEG4520) has a *q*-value of 7.4%. The other 12 proteins all have a *q*-value of 0. SAM predicts that 1 out of the 13 proteins (13 × 7.4%≅1) would be a false positive. Since MSMEG4520 has the lowest observed score d = 1.2 and a *q*-value of 7.4%, by definition, MSMEG4520 is the one most likely to be falsely called significant by the 3-fold change criterion. The *t*-test performed in Figure 3 identifies this protein as significant with a *p *value of 7.2 × e^-4 ^which is not the lowest among the proteins with greater than 3-fold change (Table 3). MSMEG4520 was originally annotated as nitrite reductase (NirA), but is recently re-annotated as sulfite reductase (SirA) [22]. SirA is essential for growth of mycobacteria on sulfite or sulfate as the sole sulfur source. It does not appear to have an apparent role in acid stress response.

**Figure 6:**
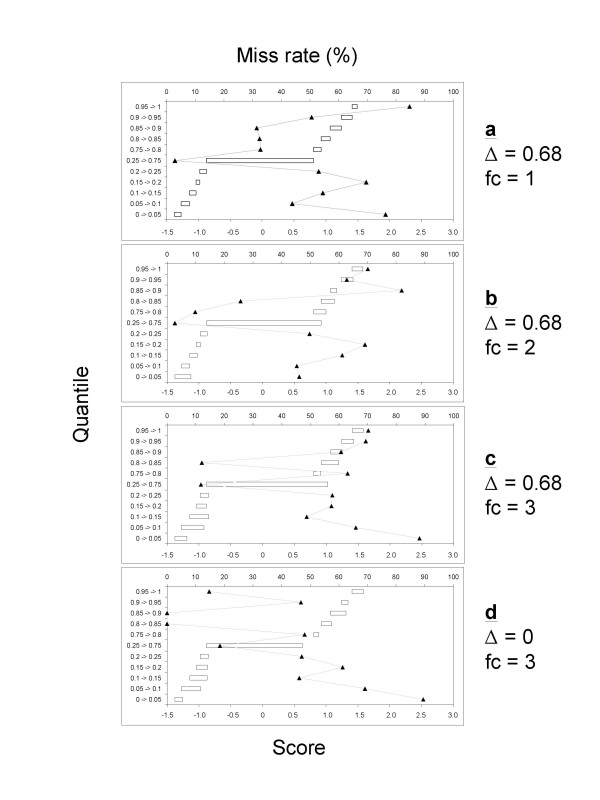
**Miss rates**. Graphic presentation of the miss rate tables for the SAM outputs shown in Figure 5. Each horizontal open bar represents the observed score cut points of a quantile of the proteins not making to the significant list. The triangle symbols represent the miss rates for the quantiles. In each panel, the left vertical category axis represents the quantiles. The top value axis represents the miss rates. The bottom value axis represents the observed scores. See text for more details.

SAM also generates a miss rate table for each Δ and/or fold change threshold. The miss rate is equivalent to a false negative rate for the proteins that are between specified score cut points and do not make the list of significant proteins. The contents of the miss rate tables for the four conditions shown in Figure 5 are presented graphically in Figure 6. The general feature is that the proteins in the 0.25–0.75 quantile range tend to have the lower miss rate, and the proteins at the two tails tend to have a higher miss rate. This is totally as expected. Comparison of panels a and b does not reveal apparent difference in the overall miss rates, suggesting that a combination of Δ = 0.68 and 2-fold change can reduce FDR without increasing miss rates compared to either Δ = 0.68 or 2-fold change alone. Thus, this combination is a more optimum criterion. Panel c shows increase in miss rates. This is expected when the 3-fold change threshold is applied in combination with Δ = 0.68. When only the 3-fold change threshold is used, the overall miss rate decreases (Panel d). The miss rate for the upregulated proteins decreases more than those for the downregulated ones. This suggests the 3-fold change threshold does not work equally for the up- and down-regulated proteins. This may be because fold change cutoff alone assumes a normal distribution, while SAM does not impose this restriction. Asymmetrical cutoff is preferred because the observed scores for up- and down-regulated proteins may behave differently in some biological experiments [15]. The samples analyzed in this study appear to be such a case.

### Differentially expressed proteins

By the 2-fold cutoff and FDR of 2.28% with Δ = 0.70 (Figure 5b), SAM found 9 induced and 12 repressed proteins in the pH5 *versus *the pH7 samples (Table 3). There were more repressed than induced proteins. This trend was similar to that observed in a microarray study of 15 min acid shocked *M. tuberculosis *by Fisher *et al*. [23], in which 20 genes were found induced while 58 were found repressed by SAM with a 1.5 fold cutoff and 2.86% FDR. Similarly, more genes were also repressed than induced when *Shewanella oneidensis *was exposed to acidic pH [24].

Of the 9 induced proteins, 2 (MSMEG1600 and MSMEG5766) are involved in purine ribonucleotide biosynthesis, 3 (MSMEG0772, MSMEG1024, and MSMEG5516) in energy metabolism, 2 (MSMEG0366 and MSMEG5709) in fatty acid and phospholipid degradation, 1 (MSMEG2382) in glutamyl-tRNA aminoacylation, and 1 (MSMEG4283) in protein degradation. Inosine-5-monophosphate (IMP) dehydrogenase (MSMEG1600;GuaB) is an important enzyme involved in guanine nucleotide synthesis, catalyzing the oxidation of IMP to xanthosine 5'-monophosphate with the concomitant reduction of NAD to NADH. The enzyme was identified as a DNA binding protein [25]. It has been reported that protein GuaB was induced by acid in *E. coli *K-12 [26], consistent with our result here that GuaB was induced in *M. smegmatis *grown at pH 5.

Genome analysis of mycobacteria has revealed an array of genes involved in lipid metabolism [27]. It has been suggested that mycobacteria grown *in vivo *are largely lipolytic rather than lipogenic due to the variety and quantity of lipids available within mammalian cells and the tubercle [28]. The acidic growth condition probably triggers induction of fatty acid degradation related proteins such as MSMEG0366 and MSMEG5709, even though there was no fatty acid supplied in the growth media for *M. smegmatis *in this study.

Cytosolic protein degradation is central to regulating various aspects of cell biology, including stress response [29]. Proteins targeted for degradation are unfolded and cleaved to release large peptides in an ATP-dependant manner. These peptides are further cleaved or degraded by endopeptidases such as aminopeptidases in an ATP-independent manner. This general scheme of cytosolic protein degradation is conserved in all organisms. While most of the enzymes involved in the upstream ATP-dependant proteolysis are more organism-specific, the enzymes involved in the downstream ATP-independent proteolysis, including leucine aminopeptidase (PepA), are present in most organisms. Induction of cytosol aminopeptidase (MSMEG4283;PepA) in pH5 grown *M. smegmatis *is consistent with the putative function of PepA in stress response.

Of the 12 repressed proteins, 2 (MSMEG3082 and MSMEG3837) have roles in biosynthesis of cofactors, prosthetic groups, and carriers. One (MSMEG3166) is an enzyme involved in central intermediary metabolism. The remaining 9 proteins are involved in energy metabolism including the ATP synthase F1 beta subunit (MSMEG4921;AtpD). Decrease in ATP synthesis and downshift of metabolism is commonly observed in cells under stressful conditions.

Schnappinger *et al*. used SAM as the significance analysis program for transcriptional analysis of adaptation by *M. tuberculosis *in phagosomal environment [30]. The results indicated that all the seven ATP synthase subunit genes (*atpBEFHAGD*) were repressed for intraphagosomal *M. tuberculosis*, consistent with the stressful condition within phagosomes. Similarly, in a gene expression analysis of *Corynebacterium glutamicum *in response to acid adaptation at pH 5.7, the seven F_0_F_1_-type ATP synthase subunits (NCgl1159-1165) were repressed [31]. In our recent study of protein turnover in *M. smegmatis *[32], AtpD was found to have lower protein turnover when logarithmically growing cells were shifted to acidic (pH5) or low iron medium, suggesting downregulation of AtpD under both stress conditions. This result further supports our finding here that AtpD was repressed in pH5 grown *M. smegmatis *cells. Since only 1 of the 5 SDS/PAGE fractions was analyzed in this study, it is reasonable to expect that other ATP synthase subunits could be found repressed as well if all the SDS/PAGE fractions were analyzed [32]. This expectation is based on the transcriptional analysis of *M. tuberculosis *and *C. glutamicum *under stress [30,31], as well as our work on protein turnover analysis of *M. smegmatis *in which three detected ATP synthase subunits (MSMEG4920, MSMEG4921, and MSMEG4926) had lower protein turnover when the *M. smegmatis *cells encountered an acidic or low iron condition [32].

## Conclusion

We have shown that the SAM method for DNA microarray data analysis can be adapted for significance analysis in LC/MS based quantitative proteomics. SAM assigns a significance value, a false discovery rate, and a miss rate for differential expression of individual proteins and groups called significant or insignificant. Such information is not readily available by conventional *t*-test or fold change test alone. The SAM method provides richer information and is more adaptive to different biological experiments that may have asymmetrical distribution of differential protein expression profiles.

One limitation of applying the SAM method for quantitative proteomics is that it requires sample replicates. Such data sets require more effort to obtain them in proteomics than in microarray analysis due to the limited MS/MS sampling speed in LC/MS analysis. In this work, we performed multiple runs for each biological replicate to cover as many proteins as possible so that enough proteins were commonly quantified between replicates. In on-going work, we will incorporate the cross-reference method that has already been developed by other research groups to align peptides between runs based on accurate mass and elution time information [6,8,9,19]. This will allow a peptide identified by MS/MS scan in one run to also be quantified in another run, even if the peptide is missed by MS/MS scan in the other run. Implementation of this cross-reference method will also make it possible to perform time course study using SAM [21]. Storey *et al*. showed that "an actual time course analysis offers a sizable increase in statistical power over a static design analysis" [33]. Measuring differential expression over time with single sampling at each time point will likely be a more sensitive study design than a typical static design even if replicates are sampled at the single time point. Once the issue of protein cross-reference between samples is addressed for quantitation of LC/MS data, it is more desirable to perform a time course study for quantitative proteomics than a single time point design with replicates. SAM is a suitable statistical analysis software for such a time course study [33].

## Methods

### Chemicals and bacterial strain

Dextrose, Tween 80, citric acid, biotin, pyridoxine, NaCl, Na_2_HPO_4_, KH_2_PO_4_, MgSO_4_·6H_2_O, CuSO_4_·5H_2_O, ZnSO_4_·6H_2_O, CaCl_2_·2H_2_O, ferric ammonium citrate, ammonium bicarbonate, and acetonitrile were purchased at certified ACS or reagent grade from Fisher Scientific (Pittsburgh, PA). 7H9 broth base and 99At% (^15^NH_4_)_2_SO_4 _were purchased from Sigma (St. Louis, MO). At% denotes atomic percent. Sequencing grade trypsin was obtained from Promega (Madison, WI). *M. smegmatis *strain mc^2 ^155 was obtained from the American Type Culture Collection (ATCC; Rockville, Md). BCA Protein Assay kit was obtained from Pierce (Rockford, IL).

### Cell culturing

Two unlabeled (i.e. ^14^N labeled) *M. smegmatis *culture samples were grown for study, one at pH 5 and the other at pH 7. Each culture sample was grown in triplicate and harvested at approximately the same OD during the exponential growth phase. During exponential growth, the cells are at the same physiological state so that the only difference is the pH value of the cultures. It is more important to ensure that cells are collected in the exponential phase rather than at the same OD [34] because cell cultures under different stresses may grow to different maximum OD. For quantitative proteomic analysis by isotope ratios, one single ^15^N labeled culture was grown as the common reference for all the replicates of the pH5 and pH7 culture samples. Since this ^15^N labeled culture was used as the reference for comparing the ^14^N labeled pH5 and pH7 cultures, we chose to collect this culture at OD 1.1 in the late exponential phase for a high cell yield.

The medium for growing the unlabeled cells was prepared with Sigma 7H9 base plus 0.05% Tween80 and 0.2% glucose. The medium pH was adjusted to 7.0 or 5.0 by titrating with 1 M sodium hydroxide or 2 M hydrochloric acid. The six unlabeled culture replicates were grown at 100 ml in loosely capped 250-ml nephelo culture flasks under shaking at 37°C. Growth was monitored by measuring turbidity in a Spec20 spectrometer (Thermo Fisher Scientific, Waltham, MA) at 600 nm. The triplicates of the pH5 culture were collected at OD 0.71, 0.69, and 0.67 and named pH5A, pH5B, and pH5C respectively. Similarly, the triplicates of the pH7 culture were sampled at OD 0.77, 0.74, and 0.76 and named pH7A, pH7B, and pH7C respectively. Only 30 ml from each culture was collected, allowing the rest of the culture to continue to grow until stationary phase for recording the complete growth curves.

The medium for growing ^15^N labeled cells consisted of (g/L) 99At% (^15^NH_4_)_2_SO_4_: 0.5; glucose: 2; Tween 80: 0.5; citric acid: 0.094; biotin: 0.0005; pyridoxine: 0.001; NaCl: 0.1; Na_2_HPO_4_: 2.5; KH_2_PO_4_: 1; MgSO_4_·6H_2_O: 0.1; CuSO_4_·5H_2_O: 0.001; ZnSO_4_·6H_2_O: 0.002; CaCl_2_·2H_2_O: 0.0007; ferric ammonium citrate: 0.04; pH5.0. The single ^15^N labeled cell culture was grown at 50 ml in a loosely capped 250-ml nephelo culture flask under shaking at 37°C. Thirty milliliter of the ^15^N labeled reference culture was collected at OD 1.1 in the late-log phase. All the collected 30 ml cultures were centrifuged at 4000 rpm in a 5810R refrigerated Eppendorf centrifuge (Fisher Scientific, Pittsburgh, PA) for 10 min at 4°C to collect the cell pellets.

### Sample preparation

Proteins were extracted from each cell pellet by bead beating using a protein extraction buffer that consisted of 100 mM ammonium bicarbonate. A protease inhibitor cocktail (Pierce) was added at 1× as recommended by manufacturer into the mixtures of cell pellet and extraction buffer during protein extraction. The mixtures were vigorously agitated for total 2 min at maximum speed in a Mini-BeadBeater™ (BioSpec, Bartlesville, OK) with 30 sec of ice cooling at the 1 min intermittent. The resulted mixtures were cleared by centrifugation at 13,000 g at 4°C for 30 min. The protein concentrations were determined with the BCA Protein Assay kit according to the standard protocol. The protein extract concentrations were 3.2, 3.2, 3.3, 3.9, 3.7, and 3.4 mg/ml for pH5A, pH5B, pH5C, pH7A, pH7B, and pH7C respectively. The concentration of the protein extract of the ^15^N labeled reference was 6.3 mg/ml.

The quantified six unlabeled protein extracts were respectively spiked with an equal amount of the ^15^N labeled reference protein extract. The six spiked protein extracts were separated by 1D-SDS/PAGE. One hundred micrograms of total proteins of a spiked protein extract was loaded for separation in each lane on a 10% Tris-HCl SDS-PAGE gel (Pierce) of 5-cm length. Gel bands were revealed by Imperial Protein Stain (Pierce) and destained overnight in water. Each lane of the gel was divided into 5 fractions. The band cutting pattern was maintained the same across all the lanes.

Only the 3^rd ^fraction from each of the lanes was processed for mass spectrometry analysis. Gel pieces were minced to 1-mm^3 ^cubes, washed, and processed for in-gel digestion and peptide extraction as previously described [35]. The final peptide extract for each spiked protein extract was concentrated to near dryness in an Eppendorf Vacufuge concentrator (Fisher Scientific) and reconstituted to 25 μl with 5% formic acid for mass spectrometry analysis as below.

### Mass spectrometry analysis and data processing

Samples were submitted for analysis at the Mass Spectrometry Laboratory of Research Resource Center at University of Illinois at Chicago. The resulted raw data files were processed with the BioWorks software (Finnigan, San Jose, CA) licensed to the facility.

The peptide extracts of all the six spiked protein extracts were analyzed by the nanoLC/LTQ-FTMS system. The LTQ-FTMS is the Finnigan hybrid mass spectrometer consisting of a linear ion trap and a Fourier transform ion cyclotron resonance instrument as a second mass analyzer manufactured by Thermo Finnigan (San Jose, CA). Each peptide extract was analyzed in triplicate runs. The instrument was operated in 24-hr unattended service mode with samples injected from an auto-sampler.

For each run, about 5 μl of peptide extract solution was loaded for separation on a 150 mm × 75 μm ZORBAX C18 reverse phase column (Agilent, Germany) with a 5–35% acetonitrile (v/v) gradient in 0.1% TFA over 60 min and detected by the LTQ-FTMS. The 60-min gradient was followed by a step-gradient elution program with 80% acetonitrile in 0.1% TFA, resulting in 90 min per run. The LTQ-FTMS was operated in a data-dependant acquisition mode with up to 10 MSMS spectra acquired after each FTMS scan. The acquired RAW data files were searched against the *M. smegmatis *strain mc^2 ^155 NCBI database in two separate searches by BioWorks, one corresponding to ^14^N labeling and the other ^15^N labeling. The precursor ion tolerance was set to ± 1.5Da and digestion enzyme was designated as trypsin with 2 missed cleavages allowed. Peptide and protein probabilities were calculated in BioWorks.

Protein quantitation procedure was based upon the previously described *QN *algorithm [10]. The program was kindly provided by Prof. Barry L. Karger's laboratory at Northeastern University and was modified in Matlab v7.2 environment to accommodate using peptide probabilities calculated by BioWorks. Relative abundance was calculated for every identified PCS [10] with *p *< 0.01. The relative abundance of a peptide is expressed as the ratio of the unlabeled sample isotopologue intensity and the ^15^N labeled reference isotopologue intensity for this peptide.

To compute the protein relative abundances, the peptide lists of replicate runs for each spiked protein extract were combined. Outliers were filtered by Dixon's Q-test (95% confidence level) before being used to calculate the protein relative abundance. Protein relative abundance refers to the ratio of the abundance of an unlabeled protein relative to that of the ^15^N labeled reference. It was calculated by averaging the qualified peptide relative abundances of a protein. Relative abundance was calculated only for proteins with at least two qualified PCS identifications [10,34]. The resulted protein relative abundances were then normalized by median.

We also generated an *in silico *pooled replicate for the pH5 and pH7 culture samples respectively. To do so, the combined peptide relative abundances for each biological replicate were first normalized by the median of these peptide relative abundances. For each sample, i.e. pH5 or pH7, the normalized peptide relative abundances from the biological triplicates were combined for computing the protein relative abundances. The protein relative abundances were finally normalized by the median of the protein relative abundances. The *in silico *pooled replicates for the pH5 and pH7 samples were named pH5p and pH7p respectively.

### Significance analysis

The significance analysis was carried out with the software Significance Analysis of Microarray (academic version 3.0 for Windows XP) obtained from Stanford University [21]. The software functions as an add-in in Microsoft Excel.

## Authors' contributions

BAPR performed cell culturing, sample processing, database search, and some data interpretation. QL performed data interpretation. Both authors read and approved the manuscript.

## Supplementary Material

Additional file 1 **Table 2 – Protein and peptide data for statistical significance analysis**. The table shows the protein relative abundance, standard deviation (SD), number of unique peptides (#Pep) and number of PCS's (#PCS) for each protein in the sample replicates pH5A, pH5B, pH5C, pH7A, pH7B, pH7C, pH5p, and pH7p. Results of the fold change test, the 2 *t*-tests, and the SAM analysis are shown to the right of the table. The '-' sign indicates missing value (for abundance data) or insignificant change (for statistical testing). The table is in Microsoft Excel format. Click here for file

Additional file 2 **Table 3 – Comparison of the SAM outputs with the conventional *t*-test results**. The first column contains the locus numbers for the locus names  with the prefix 'MSMEG' omitted for brevity. d – observed score in SAM. fc – fold change. '-' – not significant. This table is a condensed version of Table 2 showing only the regulated proteins and their statistical testing results. The table is in PDF format. Click here for file
