# Supplementary material for: Significance analysis of microarray for relative quantitation of LC/MS data in proteomics
Source: BMC Bioinformatics. 2008 Apr 10;9:187. doi: 10.1186/1471-2105-9-187 (PMC2335280; doi:10.1186/1471-2105-9-187)
Supplement: Additional file 2 — Table 3 – Comparison of the SAM outputs with the conventional t-test results. The first column contains the locus numbers for the locus names with the prefix 'MSMEG' omitted for brevity. d – observed score in SAM. fc – fold change. '-' – not significant. This table is a condensed version of Table 2 showing only the regulated proteins and their statistical testing results. The table is in PDF format. [file 1471-2105-9-187-S2.pdf]

Table 3 – Comparison of the SAM outputs with the conventional *t*-test results.

The first column contains the locus numbers for the locus names (www.tigr.org) with the prefix 'MSMEG' omitted for brevity. d – observed score in SAM. fc – fold change. '-' – not significant.

Locus no.	Protein name	SAM (see Figure 5)						*t*-test for triplicates (see Figure 4)	*t*-test for pooled (see Figure 3)
		d	fc	*q*-value(%)					
				Δ=0.70 1fc	Δ=0.70 2fc	Δ=0.70 3fc	Δ=0 3fc		
1600	inosine-5-monophosphate dehydrogenase (guaB)	4.8	2.6	2.5	0.0	-	-	6.7e-03	6.8e-21
0772	F420-dependent glucose-6-phosphate dehydrogenase (mer-2)	3.9	3.0	2.5	0.0	0.0	0.0	1.2e-02	3.4e-29
3093	glyceraldehyde-3-phosphate dehydrogenase, type I (gap)	3.6	1.8	2.5	-	-	-	1.1e-02	1.0e-199
5516	NAD-dependent aldehyde dehydrogenases	3.6	2.3	2.5	0.0	-	-	4.8e-02	1.2e-12
2382	glutamyl-tRNA synthetase (gltX)	3.5	2.8	2.5	0.0	-	-	-	2.0e-08
0829	copper/zinc superoxide dismutase (sodC)	3.2	1.9	3.8	-	-	-	1.0e-02	4.8e-13
5709	far (far)	2.9	2.2	4.4	2.3	-	-	-	2.0e-07
5224	fructose-1,6-bisphosphatase, class II (glpX)	2.6	1.6	6.3	-	-	-	3.7e-02	7.8e-16
1601	IMP dehydrogenase family protein	2.6	1.4	6.3	-	-	-	3.3e-02	1.1e-10
0219	dihydroxy-acid dehydratase (ilvD)	2.5	1.5	6.3	-	-	-	-	8.1e-05
5766	phosphoribosylformylglycinamidine cyclo-ligase (purM)	2.4	2.3	6.5	2.3	-	-	-	4.9e-03
2988.1	histidyl-tRNA synthetase (hisS)	2.4	1.5	6.5	-	-	-	-	4.5e-03
6366	UDP-galactopyranose mutase (glf)	2.3	1.2	6.5	-	-	-	-	3.0e-06
1015	probable electron transfer oxidoreductase, putative	2.3	1.2	6.5	-	-	-	4.6e-02	2.6e-11
5493	phosphoribosylaminoimidazolecarboxamide formyltransferase/IMP cyclohy	2.3	1.7	6.5	-	-	-	-	3.9e-12
4293	glutamine synthetase, type I (glnA)	2.2	1.4	6.5	-	-	-	1.5e-02	1.0e-199

0366	fadA2 (fadA2)	2.1	2.4	7.7	2.3	-	-	-	3.2e-15
5517	glucose-6-phosphate isomerase (pgi)	2.1	1.3	7.7	-	-	-	-	8.6e-10
5234	serine hydroxymethyltransferase	2.0	1.2	7.7	-	-	-	-	3.1e-40
4283	cytosol aminopeptidase (pepA)	1.9	2.0	7.7	2.3	-	-	-	4.2e-41
1024	oxidoreductase, zinc-binding (adhA)	1.8	36.7	11.5	2.3	0.0	0.0	-	1.5e-15
2377	D-3-phosphoglycerate dehydrogenase (serA)	1.8	1.6	11.5	-	-	-	-	9.1e-73
4520	nitrite reductase (nirA)	1.2	18.1	-	-	-	7.4	-	7.2e-04
5691	Luciferase-like monooxygenase superfamily	-1.5	0.51	11.5	-	-	-	-	2.8e-03
3776	argininosuccinate synthase (argG)	-1.5	0.50	9.4	-	-	-	-	6.9e-13
1651	metC (metC)	-1.7	0.73	7.8	-	-	-	-	1.8e-27
1397	translation elongation factor Tu (tuf)	-1.8	0.78	6.5	-	-	-	-	1.0e-199
3066	S-adenosylmethionine synthetase (metK)	-1.9	0.84	6.3	-	-	-	-	1.4e-55
3112	transaldolase (tal)	-2.0	0.53	5.6	-	-	-	-	4.7e-84
3837	dephospho-CoA kinase, putative	-2.1	0.44	5.0	3.0	-	-	-	3.4e-09
4297	glutamine synthetase, type I (glnA)	-2.1	0.69	5.0	-	-	-	-	2.8e-16
4905	acetyl-CoA acetyltransferase (atoB-1)	-2.2	0.54	5.0	-	-	-	-	3.3e-177
6190	glycerol kinase (glpK)	-2.3	0.17	5.0	0.0	0.0	0.0	-	9.0e-16
3166	methylmalonyl-CoA mutase, small subunit (mutA)	-2.3	0.16	5.0	0.0	0.0	0.0	-	6.1e-11
6353	propionyl-CoA carboxylase, beta subunit (pccB)	-2.3	0.72	5.0	-	-	-	-	3.6e-38
4247	3-deoxy-7-phosphoheptulonate synthase	-2.3	0.62	5.0	-	-	-	-	6.7e-03
3082	3,4-dihydrox	-2.5	0.48	4.8	0.0	-	-	-	2.6e-05
0755	adenylosuccinate synthetase (purA)	-2.5	0.71	4.8	-	-	-	4.7e-02	2.7e-07
1843	adenosylhomocysteinase	-2.6	0.70	4.8	-	-	-	4.5e-02	2.6e-12
0230	O-acetylhomoserine/O-acetylserine sulfhydrylase family protein (cysD)	-2.6	0.69	4.8	-	-	-	-	5.1e-03
3258	branched-chain amino acid ABC transporter, periplasmic	-2.7	0.57	4.8	-	-	-	3.2e-02	6.9e-85

	amino acid-bin								
5503	carbamoyl-phosphate synthase, large subunit (carB)	-2.9	0.66	4.8	-	-	-	3.2e-02	6.7e-88
3318	pyridoxal-phosphate dependent TrpB-like enzyme	-2.9	0.57	4.8	-	-	-	4.1e-02	3.5e-02
0904	isocitrate lyase (aceA)	-2.9	0.07	4.8	0.0	0.0	0.0	4.3e-02	2.1e-64
1667	succinate dehydrogenase, flavoprotein subunit (sdhA)	-3.1	0.13	4.8	0.0	0.0	0.0	4.2e-05	3.6e-17
1679	monooxygenase, putative	-3.1	0.02	4.8	0.0	0.0	0.0	3.5e-02	2.1e-39
3838	rpsA (rpsA)	-3.6	0.73	0.0	-	-	-	1.7e-02	3.8e-74
3970	lactate 2-monooxygenase	-3.6	0.12	0.0	0.0	0.0	0.0	1.7e-02	5.5e-86
0001	DNA polymerase III, beta subunit (dnaN)	-3.9	0.55	0.0	-	-	-	1.1e-02	6.1e-28
6721	glpD2 (glpD2)	-4.1	0.28	0.0	0.0	0.0	0.0	1.5e-02	2.9e-10
4921	ATP synthase F1, beta subunit (atpD)	-4.4	0.25	0.0	0.0	0.0	0.0	9.2e-03	1.9e-196
5043	malate oxidoreductase (oxaloacetatedec)	-5.4	0.28	0.0	0.0	0.0	0.0	5.1e-03	2.3e-03
6720	glycerol kinase (glpK)	-6.6	0.23	0.0	0.0	0.0	0.0	2.1e-03	1.0e-199
1842	adenosylhomocysteinase (ahcY)	-6.9	0.61	0.0	-	-	-	1.1e-03	1.6e-80
1523	DNA-directed RNA polymerase, alpha subunit	-8.4	0.71	0.0	-	-	-	2.1e-04	7.8e-96
